# Cross-Entropy Learning for Aortic Pathology Classification of Artificial Multi-Sensor Impedance Cardiography Signals

**DOI:** 10.3390/e23121661

**Published:** 2021-12-10

**Authors:** Tobias Spindelböck, Sascha Ranftl, Wolfgang von der Linden

**Affiliations:** 1 Institute of Theoretical and Computational Physics, Graz University of Technology, Petersgasse 16, 8010 Graz, Austria; spindelboeck@student.tugraz.at (T.S.); vonderlinden@tugraz.at (W.v.d.L.); 2 Graz Center of Computational Engineering, Graz University of Technology, Krenngasse 37, 8010 Graz, Austria

**Keywords:** cross-entropy, machine learning, convolutional neural network, impedance cardiography, data fusion, time-series classification, aortic pathology, aortic dissection

## Abstract

An aortic dissection, a particular aortic pathology, occurs when blood pushes through a tear between the layers of the aorta and forms a so-called false lumen. Aortic dissection has a low incidence compared to other diseases, but a relatively high mortality that increases with disease progression. An early identification and treatment increases patients’ chances of survival. State-of-the-art medical imaging techniques have several disadvantages; therefore, we propose the detection of aortic dissections through their signatures in impedance cardiography signals. These signatures arise due to pathological blood flow characteristics and a blood conductivity that strongly depends on the flow field, i.e., the proposed method is, in principle, applicable to any aortic pathology that changes the blood flow characteristics. For the signal classification, we trained a convolutional neural network (CNN) with artificial impedance cardiography data based on a simulation model for a healthy virtual patient and a virtual patient with an aortic dissection. The network architecture was tailored to a multi-sensor, multi-channel time-series classification with a categorical cross-entropy loss function as the training objective. The trained network typically yielded a specificity of (93.9±0.1)% and a sensitivity of (97.5±0.1)%. A study of the accuracy as a function of the size of an aortic dissection yielded better results for a small false lumen with larger noise, which emphasizes the question of the feasibility of detecting aortic dissections in an early state.

## 1. Introduction

**Figure 1:**
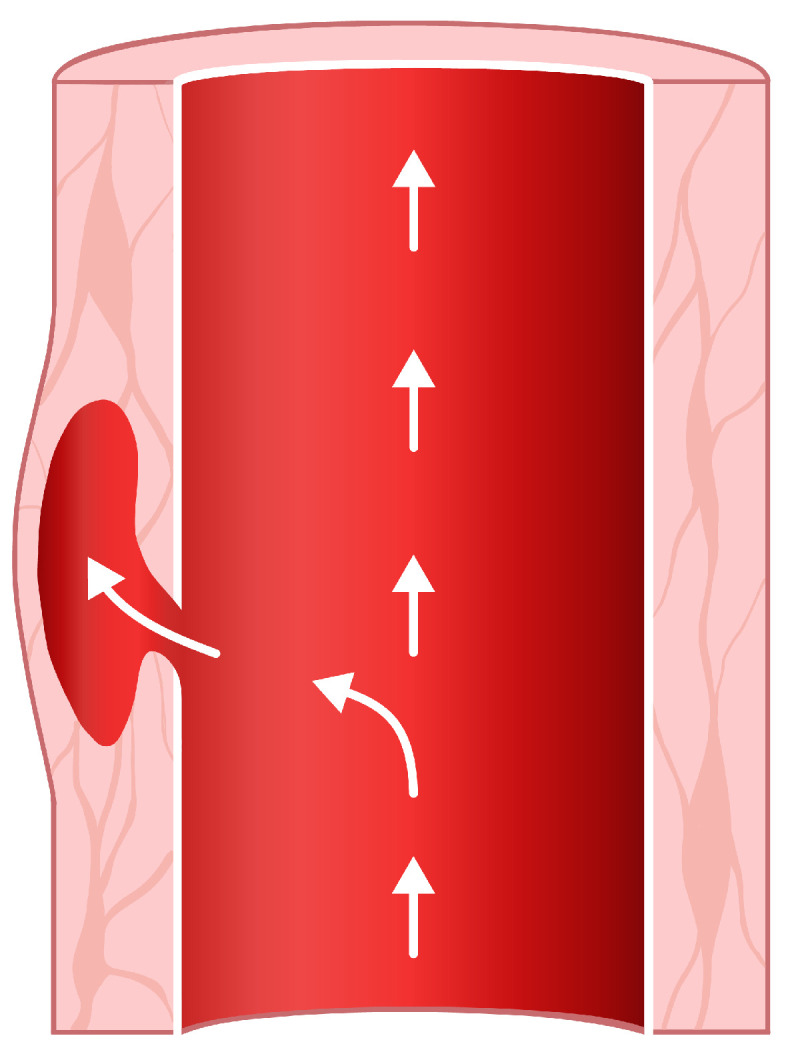
A tear in the aortic wall is filled with blood, forming a so-called false lumen.

The wall of the human aorta is a layered tissue. These tissue layers have different properties because, on the one hand, they have to be elastic and distensible as the blood is pumped through the aorta, and on the other hand, a rigid layer has to hold things together so that the aorta stays intact under periodically applied wall shear forces caused by the blood flow [1]. An aortic dissection occurs when a tear in the wall causes blood to flow between the layers (Figure 1) [1,2]. Thereby, an extra channel is formed—a so-called false lumen [1,2,3]. The presence of this false lumen, together with its entry tear and possibly an exit tear, changes the blood flow characteristics and the geometry of the aorta. Since the electrical conductivity of blood depends on the blood flow velocity, the false lumen causes a change in the cardio-synchronous component of the thoracic impedance, i.e., a change in the impedance cardiography signal (ICG) [4]. This study is limited to the particular example of aortic dissection, but the electro-physiological principles suggest that such changes in the ICG signal should occur for all aortic pathologies that change the blood flow characteristics.

**Figure 2:**
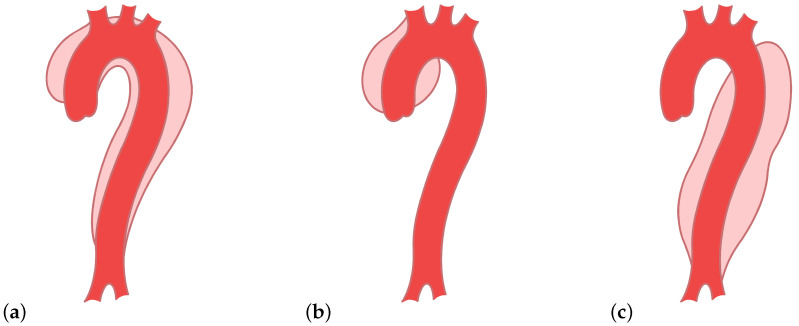
Types of aortic dissections: (**a**) DeBakey type I, (**b**) DeBakey type II, and (**c**) DeBakey type III.

The false lumen can extend along the aorta [2,5]. There are variable types of aortic dissections and two main classification systems—DeBakey types I, II, and III and Stanford A and B [5]. The type I dissection appears on the ascending and descending part, mainly near the arch, of the aorta, and the type II appears only on the ascending part [5]. Both are included in the Stanford A [1,5]. Type III, or Stanford B, involves only the descending part (Figure 2) [1,5]. These different types of aortic dissections cause different changes in the blood flow characteristics. Whether they also cause different signatures in the ICG signals is still an open question. This work is intended as a proof of concept and considers DeBakey type III (Stanford type B) aortic dissections only.

The incidence in a population of 100,000 people varies between 2 and 15 cases per year with acute aortic dissection [6,7]. Unfortunately, on average, a slight rise in cases occurred over the last decades [7]. In some cases, an aneurysm is caused due to the expansion of the false lumen [2]. Moreover, the mortality rate of acute aortic dissection of Stanford type A is around 26% during surgery and even higher with medical treatment [3]. The mortality of acute aortic dissection of Stanford type B during an emergency surgery lies between 25% and 50% [3]. An early detection would allow early treatment, effectively improving patients’ survival chances.

The state-of-the-art diagnosis methods are thoracic angiography, echocardiography, computed tomography, and magnetic resonance imaging [8,9,10,11,12,13]. These imaging methods are, however, not generally applied, and in some cases, they are not applicable at all. First, they require a trained radiologist or cardiologist, respectively. Second, MRI and CT are comparatively slow in a situation where each passing minute counts. MRI and CT are also expensive and not generally available. Angiography and echocardiography have limited imaging capabilities, i.e., the descending aorta is not visible in transthoracic echocardiography. The descending aorta is visible in transesophageal echocardiography; however, that is not a routine clinical method. Impedance cardiography (ICG) [14,15,16] was proposed as an alternative diagnostic approach [4] that is comparatively easy to use, cheap, and fast.

**Figure 3:**
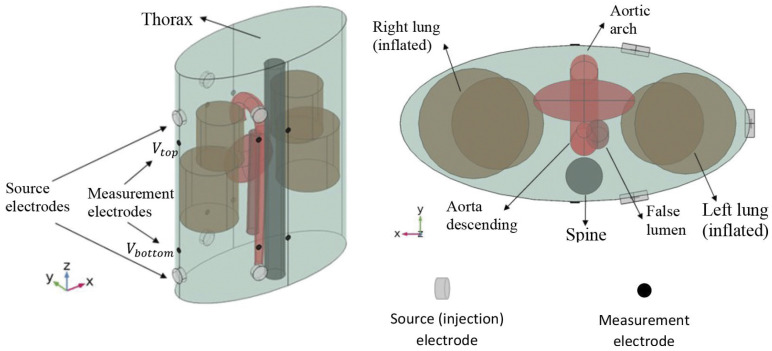
Simulated torso and placement of the injector and sensor pairs for the calculations of the artificial cardiograms. Reprinted from [4] under CC BY-NC-ND 3.0 license.

**Figure 4:**
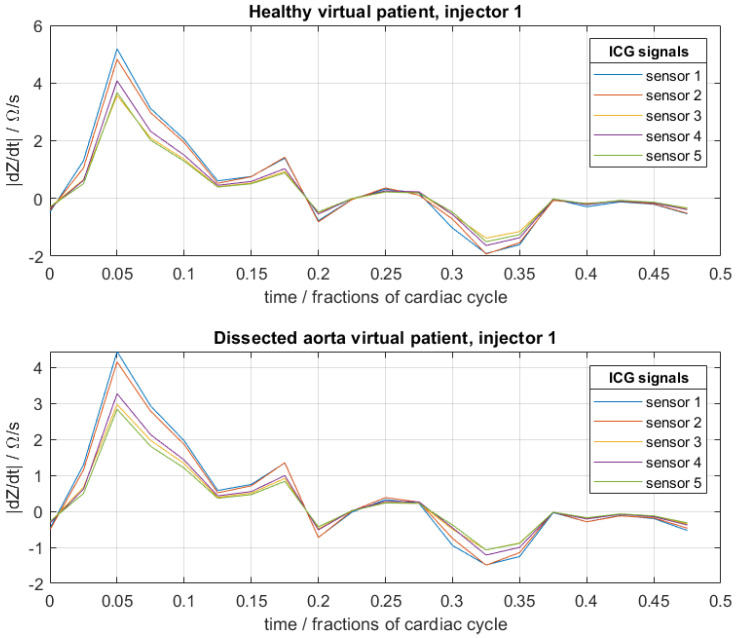
Example of artificial cardiograms of a healthy virtual patient and a virtual patient with an aortic dissection for a fixed set of physical–physiological patient-specific parameters of $R_{TL}=0.2,\;$ $R_{FL}=0.3,\;\alpha =0.8,\;\theta =0.5$ (see Section 2). For each, three such sets were generated corresponding to the three injector electrode pairs.

Here, an extension of conventional ICG to multiple sensors and injectors is considered [17]. For this, pairs of injector electrodes and pairs of sensor electrodes are placed on the thorax (Figure 3). The injector pairs are then used to apply high-frequency current with a low and constant amplitude. Because blood has a lower resistivity than the surrounding tissues, the current propagates mainly through the aorta and not through the surrounding tissues, which makes the ICG signal sensitive to changes in the blood flow. The sensor pairs measure the difference in voltage, and the impedance can be determined. Because the impedance depends on the blood flow, among other things, it changes during a full cardiac cycle, and the impedance becomes time-dependent. In the presence of an aortic dissection, the formation of a false lumen causes a small change in blood flow and volume, whereby the ICG signal also changes. These differences are hardly noticeable with the naked eye (Figure 4). In order to be able to identify such aortic dissections, a neural network is implemented in the process. The goal is to explore if it is possible to train a convolutional neural network (CNN) with artificial ICG data to detect an aortic dissection by extracting patterns in the signal, i.e., to distinguish whether a person has developed an aortic dissection or is healthy based on the impedance cardiogram [1,2,3,4,18,18].

Other approaches to detecting aortic dissections from ICG signals have been proposed via uncertainty quantification [18,19] and Bayesian model comparison [17]. These approaches have appealing analytical properties, are rigorous, and typically rely on less data. Here, we explore an alternative route that could be useful if big data are available and good scalability or a highly non-linear classifier is desired. Other related work is that of [20], who studied various machine learning algorithms (e.g., random forests and support vector machines) in combination with data pre-processing of ICG signals for the detection of valvular diseases. ICG has also been used to estimate hemodynamic parameters, e.g., cardiac output [21,22,23].

## 2. Materials and Methods

The artificial ICG data were generated via a meta-model, which was based on a simulation model provided by Badeli et al. (2020) [4]. The simulation model, as well as the meta-model, are described in detail therein. More precisely, there was one 3D finite element model (FEM) each for healthy virtual patients and virtual patients with an aortic dissection, and subsequently, there was one meta-model for each healthy and diseased virtual patient. We briefly describe here the important aspects of the simulation and the meta-model. For the simulation, three injector electrode pairs and five sensor electrode pairs were placed on the thorax, and the ICG signal was recorded for the time span of half a cardiac cycle by each sensor pair and for each injector pair. The simulation then amounted to the solution of a generalized Laplace equation on a finite element discretization of the model in Figure 3. The boundary conditions were defined by the current injection. Separate simulations were necessary for each injector pair, as this defined distinct boundary conditions. The thorax, the aorta, and other organs were modeled with simple geometrical shapes in order to reduce the problem to the salient physical–physiological effects and for reduced computation time. The current between the injector pairs was fixed with a frequency of 100 kHz and an amplitude of 5 mA.

This finite element model has been shown to be most sensitive to 4 patient-specific physiological parameters. These 4 parameters are a priori unknown in clinical practice, or are only known to lie within an interval [4]. Measuring these parameters, e.g., with the standard imaging techniques mentioned above, is not clinical practice, and would also defeat the purpose of this paper, as the discussed disadvantages apply again. We thus need the trained neural network to classify the ICG signals without precisely knowing these patient-specific parameters. These 4 patient-specific physiological parameters are (i) the maximum radius of the aorta $R_{TL}$ (radius true lumen), in the sense of the maximum over one cardiac cycle during which the elastic aorta slightly radially stretches due to dynamic pressure changes, (ii) the blood conductivity coefficient due to the hematocrit level $\theta_{H}$, (iii) the radius of the false lumen $R_{FL}$, and (iv) $\alpha_{FL}$, which denotes the angular position of the false lumen relative to the true lumen. Note that $R_{FL}$ and $\alpha_{FL}$ are not specified in the model for the healthy virtual patients. For every virtual patient, all four (two) parameters were chosen randomly between a physiologically or physically motivated minimum and a maximum. Namely, the radius of the false lumen was set between a minimum of 3 mm and a maximum of 15 mm. The hematocrit level was between 35% and 55%. The radius of the true lumen could be set between 1.35 to 1.95 cm [4]. More details on the FEM simulation are provided in [4,18]. In [4], it was shown that the radius of the false lumen had the greatest impact on the ICG data. The authors of [4,18] provided all necessary details of the assumptions and mathematical methods behind the artificial cardiograms, as well as a closer description of the different parameters.

If we would now like to create a training database of hundreds or thousands of virtual patients with random patient-specific parameters ($R_{TL},\;R_{FL},\;\alpha ,\;\theta$), this would require as many simulations. Since one finite element simulation is computationally expensive (there are no closed-form solutions), we resort to a so-called meta-model. A meta-model is a parametrized function (here, a Polynomial Chaos Expansion [24]) that is trained on examples of pairs of patient-specific parameters and corresponding full finite element simulations in order to predict ICG signals from new full simulations. The evaluation of the meta-model, once calibrated, is then computationally cheaper than the full simulation. Here, 300 finite element simulations with 300 different patient-specific parameters, as described above, were used to train the meta-model. The artificial cardiogram of a healthy virtual person or a virtual person with an aortic dissection could then be generated with the meta-models by adjusting two (healthy case) or four (diseased case) parameters. This procedure proved efficient because the meta-model approximated the simulation sufficiently accurately and reduced the computational effort for the generation of new artificial data of, in total, ca. 70,000 virtual patients (mostly for testing purposes) by several orders of magnitude.

The database for the training of the neural network was then constructed from 800 healthy and 800 diseased virtual patients. For each virtual patient, the patient-specific physiological parameters ($R_{TL},\;R_{FL},\;\alpha ,\;\theta$) were chosen randomly from a standard uniform distribution, where the interval [0,1] corresponds to the bounds described above. The data of each virtual patient consisted of the data of 3 injector electrode pairs. For each injector pair, 5 individual sensor electrode pairs measured the ICG signals over a certain time period—in this case, 20 equidistant time-steps over half a cardiac cycle. An example of the data is shown in Figure 4. The ICG signals were then predicted by the meta-model (after calibration to the full simulation) for a given set of (random) physiological parameters for each virtual patient. The meta-model allowed the prediction of ICG signals to be several orders of magnitude faster than with the original finite element model. The “noise” in these raw artificial data, due to numerical precision of each deterministic simulation (once the parameters were fixed), was negligibly small. In order to better simulate the noise levels encountered in real ICG signals, artificial zero-mean Gaussian noise with a standard deviation (std) of 0.5 was additionally superimposed on the raw data, where the noise on each data point was independent of all other data points.

For a convolutional neural network, this problem and data structure amount to a multi-sensor, multi-channel time-series classification similar to that in [25,26,27].

This database is representative for the so-constructed virtual population through the random choice of the patient-specific physiological parameters ($R_{TL},\;R_{FL},\;\alpha ,\;\theta$); however, it is not representative for a real population.

The simulation and the meta-model, respectively, do not take into account the effects of respiration or non-stationarity due to, e.g., heart rate variability. Here, we will assume a stable heart rate and that the respiratory signal components have already been separated, e.g., by principal or independent component analysis [28], ensemble averaging [29], adaptive filtering [30,31], phase-locked loop methods [32], sparse reconstruction methods [33], or Bayesian methods [34]. Whether the respiratory effects can be filtered with a neural network too remains an open question.

## 3. Cross-Entropy Loss Function

In order to train a neural network, we need a training objective, i.e., a measure to quantify how good the prediction is compared to the ground truth. This is done by using a so-called cost function or what is often referred to as an error, objective, or loss function. A typical cost function C(X,w,b) with given input $x_{n}$, *N* training examples with n={1,⋯,N}, of a dataset $X=\{x_{1},\cdots ,x_{N}\}$, weights **w**, and biases **b** of the neural net would be the mean squared error function (1) with y(x,w,b), the output of the neural network, and t(x) denotes the target output (ground truth):

(1) $$C\left(X,w,b\right)=\frac{1}{N}\sum_{n}^{N}{∥y\left(x_{n},w,b\right)-t\left(x_{n}\right)∥}^{2}$$

So, if the cost function becomes very small, the prediction error is small too. This is not necessarily true for the generalization error, but such considerations are beyond the scope of this article. Thus, minimizing the loss function with respect to weights and biases yields a set of optimal weights and biases that can later be employed to predict new data. There is a variety of possibilities for choosing a loss function or, more generally, an optimality criterion. The maximum likelihood approach is a popular principle for that. Probabilistic modeling is appealing because it introduces the notion of uncertainty. The likelihood function (2) of a distribution p(t|X,w,b,β) of independently and identically distributed variables with dependency on a known noise precision parameter β, the associated target values $t=\{t_{1},\cdots ,t_{N}\}$ being normally distributed, is given as:

(2) $$p\left(t|X,w,b,\beta \right)=\prod_{n}^{N}p\left(t_{n}|x_{n},w,b,\beta \right)$$

Taking the negative logarithm of (2) gives

(3) $$-\ln\left(p(t|X,w,b,\beta )\right)=-\sum_{n}^{N}\;\ln\;p_{n}\left(t_{n}|x_{n},w,b,\beta \right)\propto \sum_{n}^{N}{∥y\left(x_{n},w_{\mathrm{ML}},b_{\mathrm{ML}}\right)-t_{n}∥}^{2}$$

where $w_{\mathrm{ML}}$ and $b_{\mathrm{ML}}$ denote the maximum likelihood estimators given by minimizing the cost function. In (3), irrelevant prefactors are omitted and a Gaussian error is assumed. Note that we have also implicitly assumed that the likelihood is sharply peaked at the maximum likelihood estimates for the network’s weights and biases. By maximizing the log-likelihood function (3), one can get the the same weights as by minimizing a loss function (1). The two criteria, minimizing a loss function and maximizing a log-likelihood, have different values, though the optimum has the same position [35,36].

The cross-entropy is, similarly to the Kullback–Leibler divergence, a form of loss function serving as a measure of dissimilarity between two probability distributions. The cross-entropy has the form $C=-\sum_{n}^{N}t_{n}\ln\left(p_{n}\right)$; e.g., the cross-entropy between a Gaussian and a uniform distribution is the mean-squared-error function often used in least-squares regression. For *M* independent binary classifications, where m={1,⋯,M}, with a sigmoid function for the output layer, the distribution of the output variables has the form of a Bernoulli distribution. The conditional probability with the class labels $t_{m}\in \left[0,1\right]$ is then as follows:

(4) $$p\left(t|x,w,b\right)=\prod_{m}^{M}y_{m}{\left(x_{m},w,b\right)}^{t_{m}}\;{\left[1-y_{m}\left(x_{m},w,b\right)\right]}^{1-t_{m}}$$

The corresponding error-function is determined by the negative logarithm of (4) and has the form

(5) $$C\left(X,w,b\right)=-\sum_{m}^{M}\;\left[t_{m}\;\ln\;y_{m}\left(x_{m},w,b\right)+\left(1-t_{m}\right)\;\ln\left(1-y_{m}\left(x_{m},w,b\right)\right)\right]$$

This is the so-called binary cross-entropy, i.e., for binary classification problems. The cross-entropy error function accelerates training and yields better results compared to the mean-squared-error function for general classification problems [35,36]. For a problem with multi-class classification with *K* target binary output variables $t_{k}$, with k={1,⋯,K}, the error function becomes

(6) $$C\left(X,w,b\right)=-\sum_{m}^{M}\sum_{k}^{K}\;t_{mk}\;\ln\;y_{mk}\left(x_{m},w,b\right)$$

with the predicted output variables $y_{mk}\left(x_{m},w,b\right)$ for input *m* and class *k*. Equation (6) is often referred to as categorical cross-entropy. The corresponding error function (6) leads to the softmax function satisfying $\sum_{k}^{}y_{mk}\left(x,w,b\right)=1$ as an activation for the output layer. Ref. [36] (Ch. 5) suggested that there is a natural choice for selecting the output activation function related to the cost function with respect to the given problem. For regression problems, the mean-squared error with a linear output function is deemed an appropriate choice. For a multi-class classification problem, a softmax function for the output and the corresponding multi-class cross-entropy are the appropriate choice.

## 4. Neural Network Architecture for Multi-Injector, Multi-Sensor Signals

**Figure 5:**
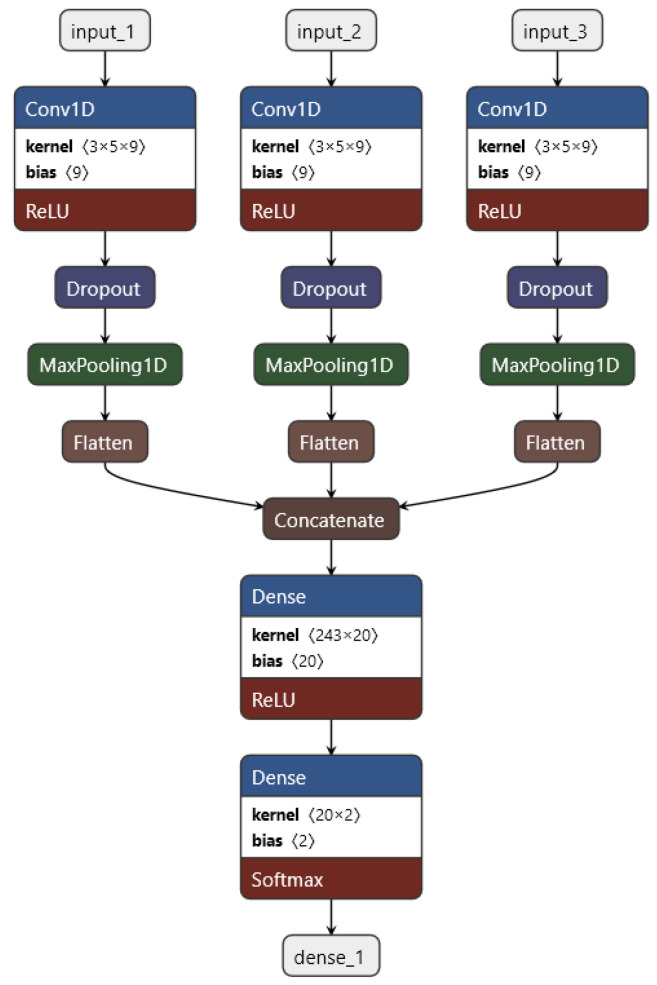
Architecture of the convolutional neural network: The data of each injector input_i is separately imported into the network. A one-dimensional convolution is performed on the data, where each sensor is seen as a channel, with a ReLu as an activation function. After that, a dropout for regularization is applied, followed by a maxpooling layer. The feature maps are flattened out into a vector, and then all three are concatenated together following a dense layer with a ReLu as activation and another dense layer for the output—two classes—with a softmax function.

Convolutional neural networks are often used in the field of image processing. Thereby, an image consists of three layers—one each for the red, green, and blue values. The two-dimensional convolution layer processes each of these layers as a separate channel [37,38,39]. A one-dimensional convolutional layer works analogously for multi-sensor time series. Here, the five sensors of one injector are each considered as a separate channel, and this would work very well, but there are three injectors altogether, and there should also be the possibility of adding the data of additional injectors in the future as well. Our approach is to treat the data of every injector separately and combine them later on after the convolution. The convolutional neural network was then programmed with Python (ver. 3.7.4) and Keras (ver. 2.4.3) with TensorFlow (ver. 2.3.1) as the back end. The complete structure of the convolutional neural network is visualized in Figure 5. The convolutional neural network has the following structure: The dataset—five time-series, one for each sensor, consisting of 20 timesteps—is imported separately for the three injectors as input_1, input_2, and input_3. Then, a one-dimensional convolution (conv1d) is performed, where the stride is 1 and a ReLu activation function is applied, whereas the five sensors are processed as five different channels for each input (in the sense of the 3 RGB-channels of images). Thereby, nine filters with a kernel size of 3 are applied. Afterwards, a dropout layer for regularization is applied in order to reduce overfitting. This is followed by a pooling layer with maxpooling and a pool size of 2. Then, the feature maps are flattened to one vector, and subsequently, all three vectors are concatenated to a single vector. The concatenated vector is fed into a dense layer with ReLu activation functions. In order to get two output neurons corresponding to the two classes “healthy” and “diseased”, an additional dense layer is integrated into the final output layer. For the output activation function, a softmax is chosen. The loss function is a cross-entropy—more precisely, a sparse categorical cross-entropy. The sparse and full categorical cross-entropy are mathematically equivalent; the only difference lies in the implementation through the data type of the labels [39].

Among a variety of advanced optimization algorithms, such as RMSProp, AdaGrad [40], or ADAM [41], we chose stochastic gradient descent (SGD) with Nesterov momentum [42] as an optimization method, which proofed to be sufficiently good for this application. The alternative adaptive methods mentioned before resulted in overfitting during the first few epochs. Additionally, the mini-batch size was 34. For a mini-batch size of 32, the network yielded slightly worse results. The learning rate was kept constant with a value of 0.09 because the neural network adapted the weights and biases too quickly, as was found empirically. As for the stopping criterion, default Keras EarlyStopping callback, set to auto and patience to 3, was integrated into the process. Thereby, the training stopped if the performance did not improve compared to the last epochs. The final convolutional network consistsed of up to 5354 trainable parameters. A summary of the hyperparameters, model choices, and other adjustable parameters of the CNN is provided in Table 1.

## 5. Results and Discussion

**Figure 6:**
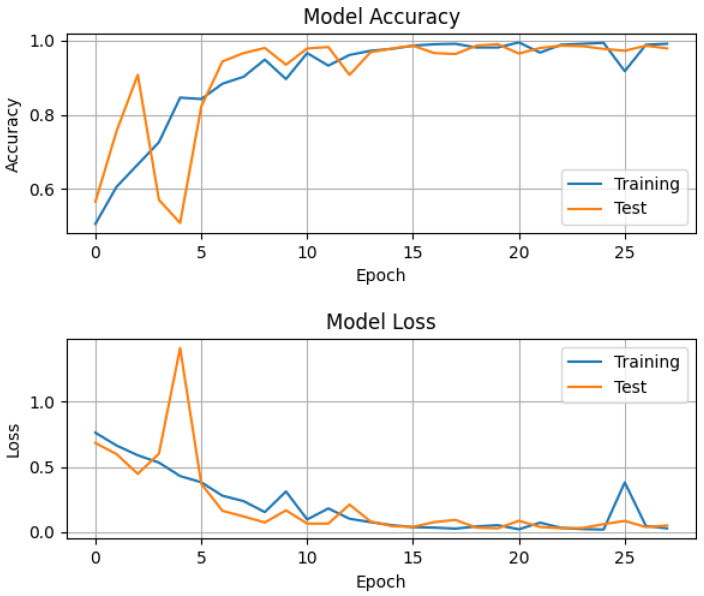
Accuracy and loss of training and test data per epoch. For both the training and test data, the four patient-specific parameters were chosen randomly for each virtual patient, and Gaussian noise with an std of about 0.5 was added. The constant learning rate is 0.09 and the optimization method was SGD with Nesterov momentum. Categorical cross-entropy was used as a loss function with a softmax function for the output.

The overall accuracy of the convolutional neural network for both classes—healthy and diseased—was around (96.6±0.1)%, as tested with the data of around 30,000 virtual patients with a ratio of 1:1 healthy and diseased, where each of the four parameters ($R_{TL},R_{FL},\alpha ,\theta$) described in Section 2 was randomly chosen for every virtual patient. On top of the artificial data, Gaussian noise with a standard deviation of 0.5 was added to model the noise in real impedance cardiography signals. The whole training process took around 10 s with an i7 laptop CPU of the 7th generation with eight threads. The accuracy, i.e., the percentage of correct predictions amongst all predictions, and the loss as defined above, for training and testing per epoch can be seen in the following graph (Figure 6).

In this figure, one can see that during the first few epochs, the accuracy and loss were oscillating as a result of a highly multi-modal cross-entropy in weight space. The initial values were generated at random. Adding Nesterov momentum to the update rule reduced these oscillations for the later epochs [43]. The system quickly approached an optimum. The order of the constant learning rate seemed to be suitable because the loss decreased quickly and then asymptotically approached a very small value close to zero.

Clinical practitioners are interested in evaluating the detector’s sensitivity and specificity to aortic dissection. For this purpose, the data of 30,000 healthy virtual patients and 30,000 aortic dissection virtual patients were generated separately. Here again, the four patient-specific parameters ($R_{TL},\;R_{FL},\;\alpha ,\;\theta$, see Section 2) were chosen randomly for each virtual patient, and Gaussian noise with an std of 0.5 was added to the data to simulate noise in real-world ICG signals. As a result, the average specificity with standard deviation was (93.9±0.1)% for a healthy virtual person. The sensitivity for a virtual patient with an aortic dissection was (97.5±0.1)%.

**Figure 7:**
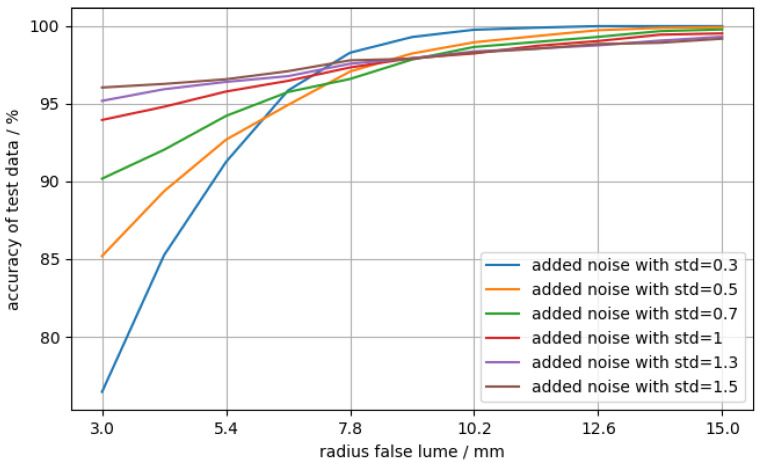
Accuracy of test data (diseased) against false lumen radius. For each data point, the cardiograms of 10,000 aortic dissection virtual patients were generated, where $R_{FL}=\mathrm{const}$, ranging from 3 to 15 mm. The three other parameters were chosen at random for each virtual patient. Gaussian noise with varying levels of standard deviation (std) as a free parameter was added on top.

A sensible cross-check is to evaluate the network’s accuracy as a function of the false lumen radius, as one would expect for the accuracy to generally increase as the false lumen grows and the magnitude of its signature in the ICG signal increases. Therefore, artificial data were generated, where the radius of the false lumen was varied between 3 and 15 mm with 11 steps. The data of 10,000 virtual patients per step were generated with always the same value for the radius of the false lumen but with random numbers for the other three parameters. Additionally, different levels of noise were added in order to evaluate the dependency of the accuracy on the noise. The result is shown in Figure 7.

In Figure 7, the accuracy of the data with stronger noise is significantly higher in the region of small false lumen radii than the cases with low noise. For larger radii, the accuracy with low-noise data has a much higher slope, whereas the accuracy of the more noisy data slightly improves. For larger radii, the accuracy is better than for smaller radii, independent of the noise level. This was expected because the magnitude of the false lumen’s signature in the ICG signal increases with the size of the false lumen [4]. Although the behavior for higher false lumen radii is as expected, the trend for small false lumen radii is surprising, as the accuracy for low-noise data is higher than for high-noise data. A possible explanation could be that the neural network learns non-obvious, non-salient features. A famous example of such an issue was discussed in [44], where a neural network trained for image classification confused a wolf with a husky due to the background (snowy or forest landscape) of the image.

By changing a few network parameters and specifications, e.g., the filter size, the learning rate, and the kernel, the overall accuracy could be improved up to 98 to 99% with the same training data and no changes to the remaining hyperparameters. This resulted in a higher specificity; however, the sensitivity with 89% was much lower than the result stated before, especially at smaller false lumen radii. The behavior seen in Figure 7 stayed mostly the same, though all of the lines were shifted downwards as a whole. This is, in principle, tolerable, as false positives do not cause harm. However, the overall probability for false positives in the general population depends on the size of the diseased population. In other words, a relatively small proportion of aortic dissections among the entire population, as indicated by their incidence, could lead to an unacceptable number of false positives. Thus, for the detector to be clinically useful, it has to have both very high sensitivity and very high specificity.

## 6. Study Limitations

For more than approximately 1000 training cases, the network tends to overfit, and for less than 400 training cases, the network clearly needs more epochs for training. The artificial data were obtained from a simplified simulation of real-world cardiograms of aortic dissections as a particular example of an aortic pathology. Other physiological effects, e.g., respiration and heart rate variability, were neglected. These might also have an important effect on the ICG signal, but, as mentioned in Section 2, here, it is assumed that these effects are filtered out. Teaching such a detector machine with real-world data might be significantly more difficult and might require modifications of the network architecture. The acquisition of a sufficient amount of such real ICG data would require elaborate clinical studies. Lastly, the interpretation of deep learning models remains difficult.

## 7. Conclusions

A convolutional neural network trained on artificial impedance cardiography data was proven to be able to detect an aortic dissection with a sensitivity of around (97.5±0.1)%, a total accuracy of around (96.6±0.1)% for both cases—diseased and healthy—and a specificity of (93.9±0.1)%. A surprising observation is the significantly better detection of aortic dissections with noisier data, as compared to low-noise data, for small false lumen radii. This behavior could be related to the difficult interpretation of deep learning models and needs further research in order to increase the accuracy and discovery rate for small false lumens. The proposed network architecture is, in principle, applicable to any classification problem with multi-sensor, multi-channel time-series data.

Lastly, the electro-physiological principles of impedance cardiography suggest that the proposed method is also applicable to other aortic pathologies that affect the blood flow characteristics. 

## Figures and Tables

**Table 1:** Summary of hyperparameters, model choices, and other adjustable parameters of the CNN.

Parameters	Values
size of training data	800
size of test data	800
size of validation data	60,000
noise added to the data	zero-mean Gaussian noise with standard deviation of 0.5
learning rate	0.09
mini-batch size	34
filters	9
kernel size	3
stride	1
activation of convolution layer	ReLu
dropout	0.2
pool size of maxpooling	2
activation of dense layer	ReLu
activation of output layer	softmax
loss function	sparse categorical cross entropy
optimization	stochastic gradient descent with Nesterov momentum
convergence criterion	default Keras EarlyStopping with patience of 3

## Data Availability

Code is available at https://github.com/user-of-relativity/CNN-artificial-impedance-cardiography (Version from 2 November 2021).
